# Granulomatous hepatitis due to *Bartonella henselae *infection in an immunocompetent patient

**DOI:** 10.1186/1471-2334-12-17

**Published:** 2012-01-23

**Authors:** Thomas R VanderHeyden, Sherri L Yong, Edward B Breitschwerdt, Ricardo G Maggi, Amanda R Mihalik, Jorge P Parada, Claus J Fimmel

**Affiliations:** 1 Department of Internal Medicine, Division of Gastroenterology, Hepatology and Nutrition, Loyola University Medical Center, 2160 South First Avenue, Maywood, IL 60153, USA; 2 Department of Pathology, Loyola University Medical Center, Maywood, IL 60153, USA; 3 Intracellular Pathogens Research Laboratory, Center for Comparative Medicine and Translational Research, College of Veterinary Medicine, North Caroline State University, Raleigh, NC 27606, USA; 4 Division of Infectious Diseases, Loyola University Medical Center, Maywood, IL 60153, USA

**Keywords:** Granulomatous hepatitis, *Bartonella henselae*, Diagnosis, Treatment

## Abstract

**Background:**

*Bartonella henselae *(*B. henselae*) is considered a rare cause of granulomatous hepatitis. Due to the fastidious growth characteristics of the bacteria, the limited sensitivity of histopathological stains, and the non-specific histological findings on liver biopsy, the diagnosis of hepatic bartonellosis can be difficult to establish. Furthermore, the optimal treatment of established hepatic bartonellosis remains controversial.

**Case presentation:**

We present a case of hepatic bartonellosis in an immunocompetent woman who presented with right upper quadrant pain and a five cm right hepatic lobe mass on CT scan. The patient underwent a right hepatic lobectomy. Surgical pathology revealed florid necrotizing granulomatous hepatitis, favoring an infectious etiology. Despite extensive histological and serological evaluation a definitive diagnosis was not established initially. Thirteen months after initial presentation, hepatic bartonellosis was diagnosed by PCR studies from surgically excised liver tissue. Interestingly, the hepatic granulomas persisted and *Bartonella henselae *was isolated from the patient's enriched blood culture after several courses of antibiotic therapy.

**Conclusion:**

The diagnosis of hepatic bartonellosis is exceedingly difficult to establish and requires a high degree of clinical suspicion. Recently developed, PCR-based approaches may be required in select patients to make the diagnosis. The optimal antimicrobial therapy for hepatic bartonellosis has not been established, and close follow-up is needed to ensure successful eradication of the infection.

## Background

Cat scratch disease (CSD) is caused by *B. henselae*, a gram-negative, aerobic alpha *Proteobacterium *that is transmitted by the bite or scratch of a cat. The traditional diagnostic criteria for CSD include (1) contact with a cat and history of a scratch or other inoculation event, (2) positive cat scratch skin test reaction, (3) regional lymphadenopathy with no other apparent etiology, and (4) characteristic histopathologic features on biopsy [1]. The development of PCR-based diagnostic assays has revealed a growing number of cases in which the traditional diagnostic criteria for CSD were absent [2].

Hepatic bartonellosis has been reported in 1-2% of CSD cases, and represents the third most common clinical manifestation after fever and lymphadenopathy. The typical symptoms include right upper quadrant pain, fevers, malaise, weight loss, chills and headaches [3].

Establishing the diagnosis of CSD-induced liver disease can be challenging:

Radiologic evaluation by contrast-enhanced CT may show a range of abnormalities, with lesions that are hypoattenuating, iso-attenuating, or show rim enhancement relative to the uninvolved liver [4]. MRI imaging may show suggestive imaging patterns of the granulomas [5].

Histologically, hepatic bartonellosis is characterized by the presence of necrotizing granulomas. They are caused by the focal accumulation of activated macrophages, with a surrounding rim of lymphocytes and fibroblasts. However, hepatic granulomas are nonspecific and may be associated with a variety of liver diseases [6]. Steiner silver staining, Bartonella-specific immunohistochemical stains, and serologic tests for Bartonella-specific antibodies [7] have been shown to have diagnostic utility but may be less sensitive and specific compared to PCR testing [8-12]. PCR assays do not rely on the presence of a humoral immune response, and may therefore be diagnostic at an earlier stage of infection, or during chronic infection in anergic patients.

Conventional bacterial cultures of liver tissue are rarely diagnostic of B. henselae hepatitis. However, Breitschwerdt and colleagues have recently developed an optimized enrichment culture method that enhances diagnostic detection and molecular typing (by DNA sequencing of the PCR amplicon) of *B. henselae *and other *Bartonella *spp. from venous blood. This highly sensitive method is able to detect active infection in patients who lack *B. henselae *antibodies and in whom conventional bacterial cultures have been negative [13,14].

In this study, we describe how the use of optimized serum and tissue PCR assays resulted in the establishment of *B. henselae *hepatitis in a young, immunocompetent patient with severe granulomatous hepatitis.

## Case presentation

In August, 2008, a 36 year-old woman with no prior history of liver disease presented to her primary care physician complaining of abdominal pain of five days duration. The pain was constant, sharp, localized to the right upper quadrant, non-radiating, and associated with nausea but no vomiting. Review of systems was positive for fatigue. Initial laboratory testing revealed only a mildly elevated alanine aminotransferase (ALT) of 42 IU/L. A right upper quadrant ultrasound demonstrated hepatomegaly and steatosis. Two weeks later, the patient presented to an outside hospital with worsening right upper quadrant pain, low-grade fevers, nausea and vomiting. An MRI of the abdomen revealed a two cm enhancing lesions of the right hepatic lobe. Fine needle biopsy of the lesion demonstrated a nonspecific, mixed inflammatory cellular infiltrate and steatohepatitis. There was no evidence of malignancy. Fungal and mycobacterial cultures were negative. The patient was discharged without a specific diagnosis, and no treatment was instituted.

**Figure 1:**
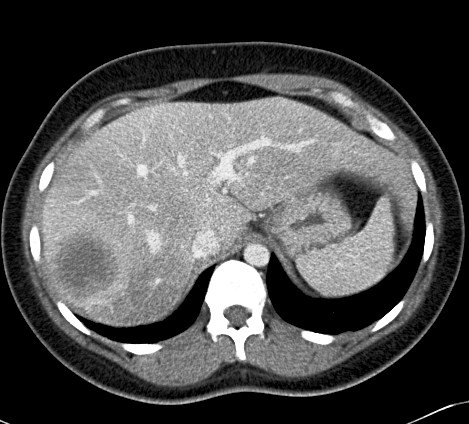
**Abdominal CT image demonstrating a large mass lesion in the right hepatic lobe**.

**Figure 2:**
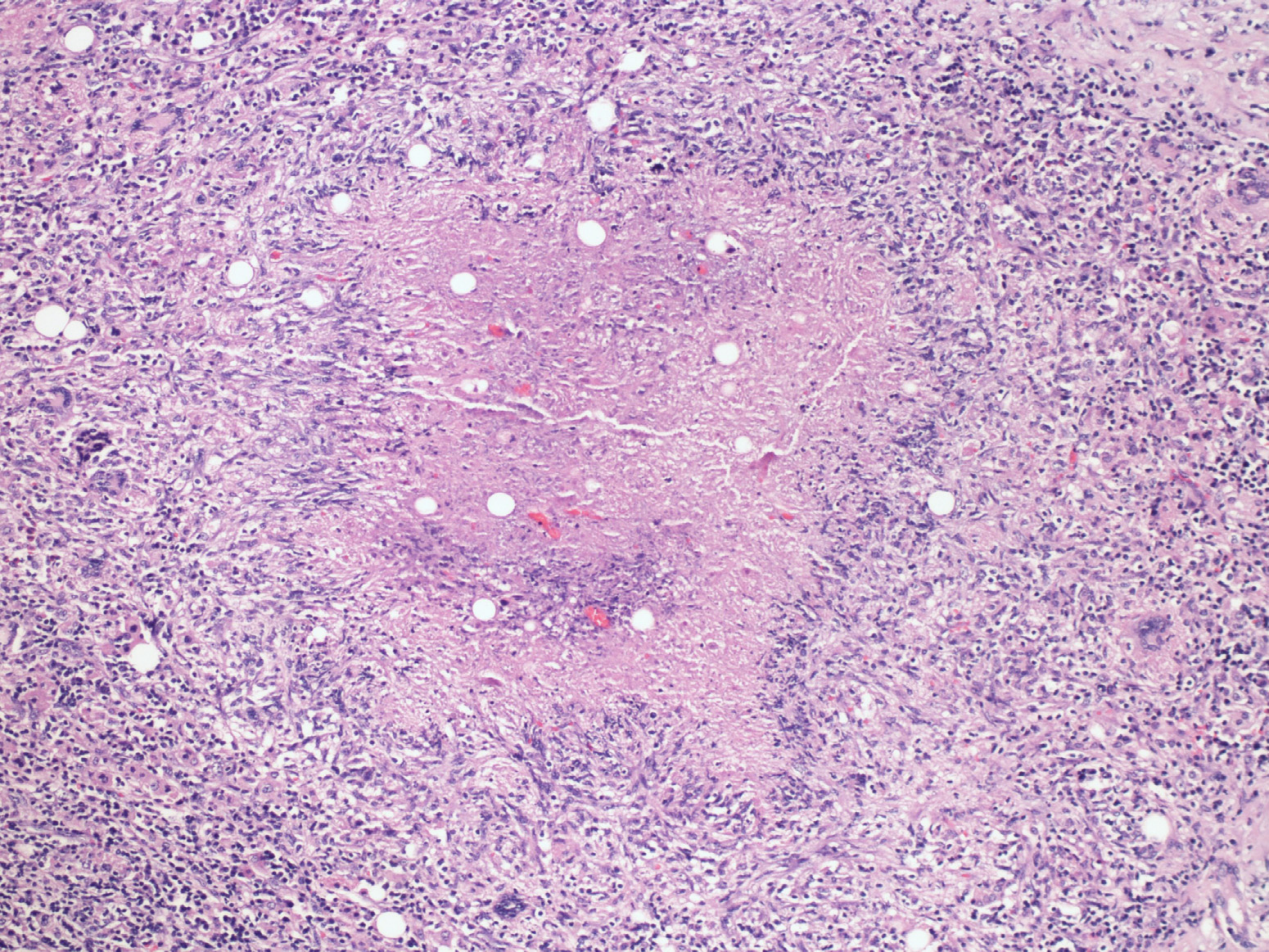
**The right partial hepatectomy shows florid necrotizing granulomatous inflammation for a peudotumorous mass (Figure 2, hematoxylin and eosin 100×)**. Necrotizing granulomatous inflammation with giant cells and characteristic palisading histiocytes.

**Figure 3:**
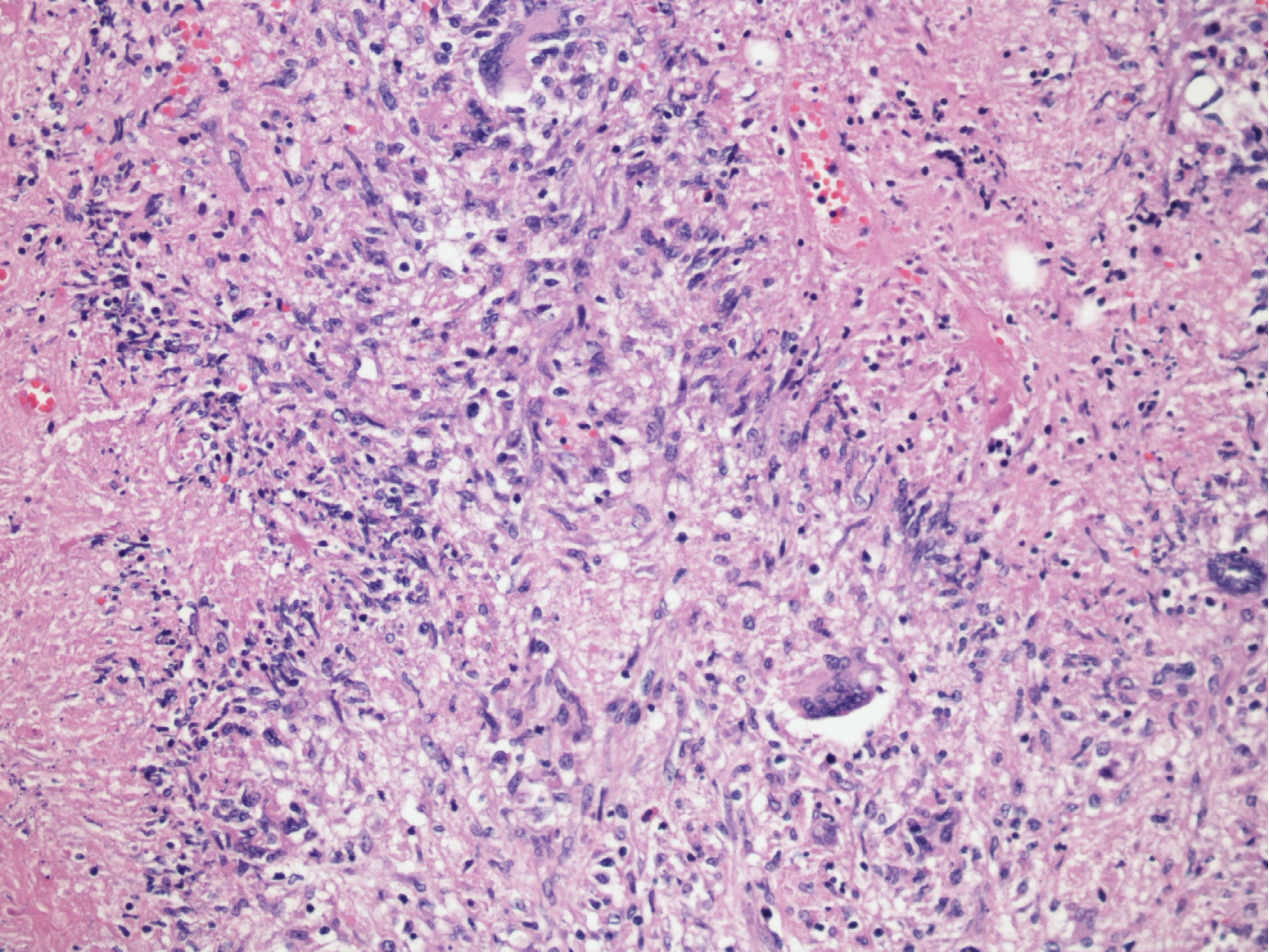
**The right partial hepatectomy shows florid necrotizing granulomatous inflammation for a peudotumorous mass**. Necrotizing granulomatous inflammation with giant cells and characteristic palisading histiocytes (Figure 3, hematoxylin and eosin 400×).

Two months later, the patient presented to Loyola University Medical Center with worsening right upper quadrant pain and fever. An abdominal CT scan revealed a 4.8 × 4.7 cm mass lesion involving the right hepatic lobe that was suspicious for malignancy (Figure 1). The patient underwent a partial right hepatectomy with excision of the mass. Histopathological studies of the resection specimen showed florid necrotizing granulomatous inflammation with pseudotumor formation (Figure 2, 3). Immuno-histochemical stains for mycobacteria, fungal organisms, and cytomegalovirus were negative. The patient's presenting symptoms resolved, and she was discharged home after an uneventful postoperative recovery.

In April of 2009, the right upper quadrant pain and low-grade fevers recurred. The patient presented to an outside hospital where an extensive evaluation was initiated (Table 1). Serologies for acute viral hepatitis were negative. Qualitative antibodies for toxoplasmosis, human immunodeficiency virus, and *Entamoeba histolytica *were negative. An EIA for *Borrelia burgdorferi *antibodies was negative. IFA assays for *Bartonella henselae *and *Bartonella quintana *were negative. Testing for urinary histoplasmosis and blastomycosis antigens and cryptococcus serum antigens were negative. Skin testing for tuberculosis and quantiferon gold were negative. A random liver biopsy showed mild macro- and micro-vesicular steatosis and non-specific chronic inflammation. Repeat special stains, fungal and mycobacterial cultures were negative. The patient's symptoms improved, and she was discharged home on analgesics.

**Table 1:** Serological tests performed to identify the etiology of granulomatous hepatitis in the patient

Test	Reference Range	Result Admission 1	Result Admission 2
Antibody to human immunodeficiency virus	Negative	Negative	Negative

Quatiferon Gold	Negative	Negative	

Epstein Barr virus			

IgM	Negative	Negative	

IgG	Negative	Positive	

Bartonella Antibody			

*B. quintana*-IgM, IgG	Negative at 1:20, 1:60 dilution	Negative	Negative

*B. hensalae*		Negative	

Lyme Antibody	Negative	Negative	

Toxoplasma			

IgM	Negative	Negative	

IgG	Negative	Negative	

Rapid plasma reagin	Negative		Negative

Entamoeba Antibody	Negative	Negative	

*Coxiella bruniti *(ELISA)	Negative		Negative

Brucella Antibody			

IgM	Negative at 1:20 dilution		Negative

IgG	Negative at 1:60 dilution		Negative

Cyrptococcal Ag (serum)	Negative	Negative	

Histoplasma Ag (urinary)	Negative	Negative	

Blastomycosis Ag (urinary)	Negative	Negative	

**Figure 4:**
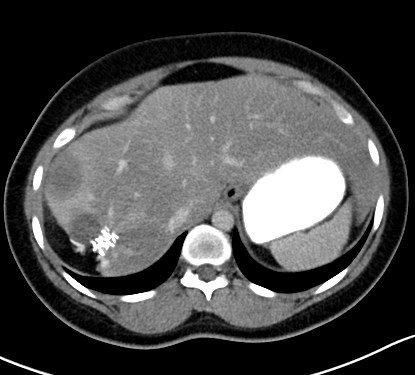
**Repeat abdominal CT after initial hepatic resection**. Several new, low-attenuation lesions are present in the right hepatic lobe. Similar, smaller lesions were present in the left hepatic lobe (not shown).

In July of 2009, the patient once again presented to an outside hospital with right upper quadrant pain, nausea, and fevers of up to 102.^4^°F. An abdominal CT revealed several low attenuation lesions involving both segments of the liver, with the largest one measuring 4.1 × 2.9 cm (Figure 4). The patient was transferred to Loyola University Medical Center for further evaluation. A detailed travel and exposure history to ticks, rodents or other vectors of unusual infectious etiologies was unrevealing. On physical examination, the patient was afebrile. There was no lymphadenopathy or hepato-splenomegaly. Her right upper quadrant was tender to palpation without rebound tenderness or guarding. Laboratory analysis revealed an aspartate aminotransferase (AST) of 93 IU/L UNITS, alanine aminotransferase (ALT) of 74 IU/L, alkaline phosphatase of 193 IU/L, and total bilirubin of 0.6 mg/dL. The hemoglobin was 13.4 gm/dL, and the WBC count was 4.9 K/UL with a normal differential. Bacterial blood cultures, urinalysis, stool cultures, and stool tests for *Clostridium difficile *were negative. A chest radiograph showed no infiltrates or lymphadenopathy. The antinuclear antibody was 1:40, and anti-mitochondrial antibody testing was negative.

On the third day of her hospitalization, the patient developed a temperature of 101.^1^°F. A comprehensive investigation for fever of unknown origin was initiated (see Table 1). Antibody titers for *Bartonella quintana*, and *Brucella *sp. were negative. An RPR was negative. Repeat HIV antibody testing was negative. A peripheral smear for malaria was negative. A transesophageal echocardiogram did not reveal any valvular vegetations. The patient refused a lumbar puncture. A percutaneous liver biopsy was performed and revealed extensive granulomatous hepatitis with occasional fibrin rings, with a background of mixed, micro- and macro-vesicular steatosis. Based on the presence of fibrin rings, the possibility of Q fever was entertained. However, ELISA testing for *Coxiella burnetti *antibodies was negative. Repeat special stains and cultures for AFB, fungal organisms, and cytomegalovirus were negative. Steiner silver stains for spirochetes and bacteria - including *Bartonella *sp.- were negative. The patient was empirically treated with a seven-day course of piperacillin and tazobactam. She remained afebrile for the remainder of her hospitalization. Based on the diagnosis of granulomatous hepatitis and the unrevealing workup for infectious organisms, the patient was started on empiric prednisone. Her liver enzymes, which had already been down-trending at the start of prednisone treatment, normalized over the next several weeks. Her symptoms resolved, and she was discharged home on a tapering dose of prednisone.

Based on the striking features of her hepatic granulomas, the possibility of hepatic Bartonella infection was raised, despite the negative Bartonella antibody titers. A sample of formalin-fixed liver tissue from the initial liver resection was sent to the University of Arkansas for PCR testing. Using previously validated assay conditions, a 153 bp fragment of the *B. henselae *16S rRNA gene was amplified, and confirmed by Southern blot hybridization [15,16]. Upon further questioning, the patient reported that she had intermittently come in contact with a cat while visiting her mother's house, although she did not recall any cat scratches or bites.

Based on the presumptive diagnosis of hepatic bartonellosis, the patient was started on azithromycin 250 mg daily, and her prednisone was discontinued. Two weeks later, the patient developed diarrhea and abdominal cramping that were attributed to her antibiotic. Azithromycin was discontinued, and the patient was started on a nine-week course of clarithromycin at a dose of 500 mg twice daily. Her symptoms completely resolved.

In May of 2010, the patient presented to our emergency room with recurrent right upper quadrant pain. On examination, she was afebrile and her vital signs were stable. There was no jaundice. Right upper quadrant tenderness was present in the area of the excisional scar. There was no palpable hepato-splenomegaly. No skin rashes were present. AST and ALT levels were elevated to 109 IU/L and 75 IU/L, respectively. The total bilirubin, alkaline phosphatase, and INR were normal. A liver-protocol CT revealed a 2.4 cm, low-density lesion at the previous surgical site, which was thought to be nonspecific. However, given her recurrent symptoms, she was empirically treated with a six-week course of ciprofloxacin 500 mg twice daily.

**Figure 5:**
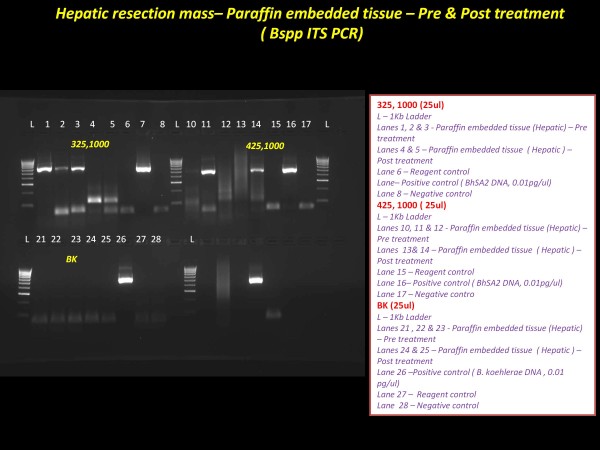
**PCR results targeting the *Bartonella *16S-23S rRNA intergenic spacer region using primers 325-1000 and primers 425-1100 (top row) and *Bartonella koehlerae *specific primers (bottom row)**. With primers 325-1000, amplicons were obtained in lanes 1-3 from the resected liver lobe (pre-antibiotic treatment) but not in Lanes 4-5 (post-treatment liver biopsy). With primers 425-1000, single amplicons were obtained in the pre- (Lane 2) and post-treatment (Lane 5) liver samples. By DNA sequencing, all amplicons corresponded to a 16S-23S strain of *B. henselae*. No amplicons were obtained with the *B. koehlerae *primer set. RC = DNA extraction control, lane, BH = *B. henselae *(Houston I strain), PO = PCR negative control, M = Kb DNA ladder.

At a subsequent clinic visit in September of 2010, she reported improvement in her abdominal pain. However, the AST and ALT activities had risen to 139 IU/L and 195 IU/L, respectively. A transjugular liver biopsy demonstrated small, scattered, non-caseating granulomas with a background of micro- and macrovesicular steatosis. A sample from the biopsy was sent to the University of Washington for PCR analysis. Using conventional PCR conditions and primers targeting the ribC gene (5'-GATATCGGTTGTGTTGAAGA-3', 5'-AATAAAAGGTATAAAACGCT-3') [17], a 393 bp PCR product specific for *B. henselae *was amplified from the biopsy material. In order to determine whether the patient was bacteremic, venous blood samples were sent to the Intracellular Pathogens Research Laboratory (IPRL), Center for Comparative Medicine and Translational Research, College of Veterinary Medicine, North Carolina State University. Using a previous described diagnostic platform [13,14], conventional PCR targeting the 16S-23S intergenic spacer (ITS) region was performed on DNA extracted from blood, serum, and *Bartonella *alpha Proteobacteria growth medium (BAPGM)enrichment blood cultures. No amplification products were obtained from pre-enrichment blood and serum samples obtained on three sequential days. In contrast, a target band was obtained from one of three post-enrichment blood cultures. DNA sequencing of the amplicon was diagnostic for the ITS region of the SA2 strain of *B. henselae*, thereby establishing *B. henselae *bacteremia. The patient's serum tested negative for antibodies against *B. henselae, B. koehlerae*, or *Bartonella vinsonii *subsp. *berkhoffii *genotypes I, II or III antigens [14]. Subsequently, using 16S-23S ITS primers, *B. henselae *DNA (SA2 strain by DNA sequencing) was amplified and sequenced from paraffin blocks containing material from the initial liver resection (January 2009) and from the follow-up biopsy obtained in September of 2010 (Figure 5). These data demonstrated the presence of the SA2 *B. henselae *strain in the patient's liver throughout her course. Immunohistochemical staining for *B. henselae *using a recently described *B. henselae *monoclonal antibody assay [18] showed rare coccobacilli with positive staining and appropriate morphology for *B. henselae*, however, clusters of organisms were not visualized (data not shown).

## Conclusions

Our case illustrates the unique challenges of establishing and treating *B. henselae*-induced granulomatous hepatitis.

With regard to the establishment of the diagnosis, many of the typical clinical and laboratory features of CSD were absent. Similar to several case reports in the literature, our patient presented with atypical and nonspecific constitutional symptoms [19]. Steiner silver stains of infected liver tissue and immunohistochemical assays did not reveal the culprit organism, and serologic tests failed to detect *B. henselae*-specific antibodies. A high degree of clinical suspicion, persistent attempts to establish a specific diagnosis, and the use of optimized enrichment cultures and PCR amplification were ultimately required to establish the diagnosis in our patient. Strikingly, PCR assays targeting three distinct sequences and performed at three different laboratories confirmed the presence of *B. henselae *at multiple time points in the patient's liver and serum, suggesting that this bacterium was the likely causative agent in this case.

With respect to treatment options, our patient's course challenges the traditional view of CSD as an inevitably self-limited disease. Recent guidelines have stressed the importance of prolonged treatment and antibiotic combination therapy in subgroups of patients with CSD, including individuals with HIV infection, bacillary angiomatosis, peliosis hepatis, retinitis, chronic bacteremia, endocarditis, chronic lymphadenopathy and neurological disorders [20]. However, prospective studies validating these recommendations are lacking.

In vitro testing has demonstrated sensitivity of *B. henselae *strains to a wide spectrum of antibiotics including beta-lactams, macrolides, cephalosporins, aminoglycosides, flouroquinolones, doxycycline and rifampin [21]. However, most of these agents are bacteriostatic and fail to eliminate the bacterium when used as monotherapy. In the only prospective, double-blind, placebo-controlled study on immunocompetent patients with CSD [22], azithromycin monotherapy resulted in improved lymphadenopathy but did not prevent dissemination or infectious complications. This disappointing result may have been due to the development of azithromycin resistance *by B. henselae *[23]. Recurrent bacteremia has been described in several reports [24], even after a prolonged antibiotic course.

Our review of the published literature identified a small number of retrospective studies using antibiotic combination therapy: Arisoy and colleagues demonstrated the efficacy of rifampin and gentamycin in a small cohort of pediatric patients [25]. In a single case report, the triple combination of doxycycline, erythromycin and azithromycin cleared *B. henselae*-induced, post liver-transplant granulomatous hepatitis [26,27]. A combination of doxycycline (100 mg twice daily) and rifampin (300 mg twice daily) was used successfully to treat *B. henselae *retinitis [28]. Prednisone has been suggested as adjunctive therapy in patients with antibiotic-refractory hepatic bartonellosis. However, immunosuppression should be used with caution, as it might contribute to the development of Bartonella endocarditis [29,30].

Our patient was initially treated with surgical resection, reminiscent of a previous case report by Murano in which a giant, B. henselae-induced granuloma in a 10-year old child required a partial hepatectomy [31]. This was followed by multiple courses of empiric antibiotic therapy, including piperacillin/tazobactam, azithromycin, clarithromycin, and ciprofloxacin. None of these treatments eradicated the infection. Following the establishment of *B. henselae *bacteremia and the demonstration that *B. henselae *genetic material was present in the patient's liver throughout the course of her disease, we opted to treat the patient with an 8-week course of rifampin (300 mg bid) and doxycycline (100 mg bid). Three months following this most recent treatment, the patient's liver enzymes have completely normalized for the first time, and her constitutional symptoms have not recurred. We are planning on a long-term follow-up and repeat enrichment blood cultures in the future.

## Informed consent

Written informed consent was obtained from the patient for publication of this case report and any accompanying images. A copy of the written consent is available for review by the Editor-in-Chief of this journal.

## Competing interests

The authors declare that they have no competing interests.

## Authors' contributions

TV participated in the clinical care of the patient and wrote the manuscript. SY carried out the pathologic studies. EB participated in the planning of the molecular studies and editing of the final manuscript. RM performed molecular analyses including PCR and DNA sequencing. AM participated in the initial evaluation and treatment of the patient. JP assisted in the selection of anti-microbial therapy. CF served as the primary hepatologist and edited the manuscript. All authors read and approved the final manuscript.

## Pre-publication history

The pre-publication history for this paper can be accessed here:

http://www.biomedcentral.com/1471-2334/12/17/prepub
